# COVID-19 Vaccination and Odds of Post–COVID-19 Condition Symptoms in Children Aged 5 to 17 Years

**DOI:** 10.1001/jamanetworkopen.2024.59672

**Published:** 2025-02-24

**Authors:** Anna R. Yousaf, Josephine Mak, Lisa Gwynn, Karen Lutrick, Robin F. Bloodworth, Ramona P. Rai, Zuha Jeddy, Lindsay B. LeClair, Laura J. Edwards, Lauren E.W. Olsho, Gabriella Newes-Adeyi, Alexandra F. Dalton, Alberto J. Caban-Martinez, Manjusha Gaglani, Sarang K. Yoon, Kurt T. Hegmann, Andrew L. Phillips, Jefferey L. Burgess, Katherine D. Ellingson, Patrick Rivers, Jennifer K. Meece, Leora R. Feldstein, Harmony L. Tyner, Allison Naleway, Angela P. Campbell, Amadea Britton, Sharon Saydah

**Affiliations:** 1 Coronavirus and Other Respiratory Viruses Division, National Center for Immunization and Respiratory Diseases, Centers for Disease Control and Prevention, Georgia; 2 Department of Pediatrics, Miller School of Medicine, University of Miami, Miami, Florida; 3 Department of Public Health Sciences, Miller School of Medicine, University of Miami, Miami, Florida; 4 Department of Family and Community Medicine, College of Medicine, University of Arizona, Tucson; 5 Abt Associates, Rockville, Maryland; 6 Section of Pediatric Infectious Diseases, Department of Pediatrics, Baylor Scott & White Health and Baylor College of Medicine, Department of Medical Education, Texas A&M University College of Medicine, Temple; 7 Rocky Mountain Center for Occupational and Environmental Health, Department of Family and Preventive Medicine, University of Utah Health, Salt Lake City; 8 Department of Community, Environment and Policy, Mel and Enid Zuckerman College of Public Health, University of Arizona, Tucson; 9 Department of Epidemiology and Biostatistics Department, Mel and Enid Zuckerman College of Public Health, University of Arizona, Tucson; 10 Marshfield Clinic Research Institute, Marshfield, Wisconsin; 11 St Luke’s Regional Health Care System Infectious Disease Associates, Duluth, Minnesota; 12 Kaiser Permanente Center for Health Research, Portland, Oregon

## Abstract

**Importance:**

An estimated 1% to 3% of children with SARS-CoV-2 infection will develop post–COVID-19 condition (PCC).

**Objective:**

To evaluate the odds of PCC among children with COVID-19 vaccination prior to SARS-CoV-2 infection compared with odds among unvaccinated children.

**Design, Setting, and Participants:**

In this case-control study, children were enrolled in a multisite longitudinal pediatric cohort from July 27, 2021, to September 1, 2022, and followed up through May 2023. Analysis used a case (PCC reported)–control (no PCC reported) design and included children aged 5 to 17 years whose first real time–polymerase chain reaction (RT-PCR)–confirmed SARS-CoV-2 infection occurred during the study period, who were COVID-19 vaccine age-eligible at the time of infection, and who completed a PCC survey at least 60 days after infection. From December 1, 2022, to May 31, 2023, children had weekly SARS-CoV-2 testing and were surveyed regarding PCC (≥1 new or ongoing symptom lasting ≥1 month after infection).

**Exposures:**

COVID-19 mRNA vaccination status at time of infection was the exposure of interest; participants were categorized as vaccinated (≥2-dose series completed ≥14 days before infection) or unvaccinated. Vaccination status was verified through vaccination cards or vaccine registry and/or medical records when available.

**Main Outcome and Measures:**

Main outcomes were estimates of the odds of PCC symptoms. Multivariate logistic regression was performed to estimate the odds of PCC among vaccinated children compared with odds of PCC among unvaccinated children.

**Results:**

A total of 622 participants were included, with 28 (5%) case participants and 594 (95%) control participants. Median (IQR) age was 10.0 (7.0-11.9) years for case participants and 10.3 (7.8-12.7) years for control participants (*P* = .37). Approximately half of both groups reported female sex (13 case participants [46%] and 287 control participants [48%]). Overall, 57% of case participants (16 children) and 77% of control participants (458 children) were vaccinated (*P* = .05). After adjusting for demographic characteristics, number of acute COVID-19 symptoms, and baseline health, COVID-19 vaccination was associated with decreased odds of 1 or more PCC symptom (adjusted odds ratio [aOR], 0.43; 95% CI, 0.19-0.98) and 2 or more PCC symptoms (aOR, 0.27; 95% CI, 0.10-0.69).

**Conclusions and Relevance:**

In this study, mRNA COVID-19 vaccination was associated with reduced odds of PCC in children. The aORs correspond to an estimated 57% and 73% reduced likelihood of 1 or more and 2 or more PCC symptoms, respectively, among vaccinated vs unvaccinated children. These findings suggest benefits of COVID-19 vaccination beyond those associated with protection against acute COVID-19 and may encourage increased pediatric uptake.

## Key Points

**Question:**

Does COVID-19 mRNA vaccination reduce the occurrence of post–COVID-19 condition (PCC) following SARS-CoV-2 infection in children aged 5 to 17 years?

**Findings:**

In this case-control study with 622 participants, vaccination was associated with a 57% decreased odds of 1 or more PCC symptoms and a 73% decreased odds of 2 or more PCC symptoms.

**Meaning:**

The findings of this study suggest that mRNA COVID-19 vaccination may be a protective factor against PCC in children following SARS-CoV-2 infection.

## Teaser

This case-control study among US children estimates the association of mRNA COVID-19 vaccination with likelihood of post–COVID-19 condition.

## Introduction

An estimated 1% to 3% of children infected with SARS-CoV-2 will develop post–COVID-19 condition (PCC).^1,2^ Because millions of children in the United States and worldwide have been infected with SARS-CoV-2, PCC in children is a critical research area.^3^ Although children typically experience mild symptoms from SARS-CoV-2 infection, PCC can develop following mild or severe COVID-19 illness, and PCC symptoms can be prolonged, debilitating, and contribute to school absenteeism.^3,4,5^

PCC, also known as long COVID, long-haul COVID, or postacute sequelae of SARS-CoV-2, is a group of diagnoses and symptoms following SARS-CoV-2 infection that were first recognized in adults.^4^ PCC has since been recognized in children but remains less well understood, as the majority of PCC research has focused on adults. There are several similar PCC definitions^6,7^ as well as one recently published by the National Academies of Sciences, Engineering, and Medicine (NASEM) which defines PCC as a “chronic condition that… is present for at least 3 months as a continuous, relapsing and remitting, or progressive disease state that affects one or more organ systems.”^8^ This study was conducted before publication of the NASEM definition and uses the PCC definition of signs, symptoms, and conditions that continue or develop after initial COVID-19 disease or SARS-CoV-2 infection that are present 4 or more weeks after the initial phase of infection.^9^

The pathophysiology behind PCC remains poorly understood and is likely multifactorial.^10^ Signs and symptoms of PCC in children are similar to those in adults and include symptoms affecting multiple organ systems, variable combinations of symptoms, and symptoms that may progress over time.^1,4^ PCC symptoms most commonly identified in children include respiratory symptoms, fatigue, weakness, exertional malaise, change in energy level, mood changes, and sleep disturbance.^4,11,12^ PCC also includes serious and life-threatening diagnoses, such as thrombotic events (acute pulmonary embolism, venous thromboembolism), myocarditis, cardiomyopathy, kidney failure, and type 1 diabetes.^13,14^ Risk factors for PCC in children include severe COVID-19 illness and hospitalization, older age (6-18 years), and underlying medical conditions.^15,16,17^

Given the potential number of children infected by SARS-CoV-2 and therefore at risk of developing PCC, understanding factors that reduce the occurrence and severity of PCC is essential. While data from adult studies show that COVID-19 vaccination is associated with a reduced risk of PCC,^18,19,20^ data on the impact of COVID-19 vaccination on PCC in children are limited, with 1 study showing no difference in PCC between vaccinated and unvaccinated children,^21^ another showing a reduced risk of PCC associated with vaccination that was not statistically significant,^15^ and 2 others showing moderate protection from COVID-19 vaccination against PCC.^22,23^ The aim of this study is to assess the association of COVID-19 vaccination with PCC in a cohort of children by estimating the odds of PCC among children who were vaccinated compared with the odds of PCC among those who were unvaccinated.

## Methods

### Study Population

Participants were enrolled from the Pediatric Research Observing Trends and Exposures in COVID-19 Timelines (PROTECT) study, a previously described longitudinal prospective SARS-CoV-2 surveillance cohort established in July 2021.^24^ In brief, children aged 6 months to 17 years were enrolled at 4 US study sites: Florida (Miami), Texas (Temple), Utah (Salt Lake City), and Arizona (Phoenix, Tucson, and other areas). Participants for this analysis were enrolled from July 27, 2021, through September 1, 2022, and followed up through May 2023. Written consent from guardians and written or verbal assent from children were obtained per site protocols. PROTECT was reviewed by the US Centers for Disease Control and Prevention (CDC) and approved by the institutional review boards at University of Arizona and Abt Associates under reliance agreements; the study was conducted consistent with applicable federal law and CDC policy. This study followed the Strengthening the Reporting of Observational Studies in Epidemiology (STROBE) reporting guidelines.^25^

### Data Collection

All enrolled children provided weekly self- or guardian-collected nasal swabs for SARS-CoV-2 screening via real time–polymerase chain reaction (RT-PCR) test. Participants with a positive RT-PCR test or reported COVID-19–like illness (CLI) were sent weekly illness surveys until symptoms (if any) resolved. Dates of positive in-study tests ranged from December 20, 2021, through March 7, 2023, and illness surveys were completed December 27, 2021, through April 10, 2023. Information on demographic characteristics, self-described health status (excellent, very good, good, fair, or poor), underlying health conditions, daily medication use, and COVID-19 vaccination status was collected from participants or their guardians at enrollment and updated at regular intervals. A subset of positive RT-PCR tests was sent for sequencing. History of SARS-CoV-2 infection before study enrollment was determined via self-report with documentation of a positive test or presence of SARS-CoV-2 antibodies at enrollment (before any in-study SARS-CoV-2 infection or vaccination) for participants who provided an optional blood specimen. Participants with self-reported history of infection without documented test results and those with a positive serology test obtained after COVID-19 vaccination and no other documentation of infection were treated as not having evidence of infection before enrollment (because serology testing did not distinguish anti-spike from anti-nucleocapsid antibodies). A survey on PCC was sent out to all children with a positive in-study RT-PCR test to be completed 60 or more days after their first in-study positive test (eMethods 2 in Supplement 1). The survey asked about new or ongoing symptoms lasting at least 1 month from the referenced positive SARS-CoV-2 test. Participants were asked about symptom duration and impact on function (assessed by asking whether PCC symptoms “reduced ability to carry-out day-to-day activities, including attending school and participating in activities and sports?” with answer options of “Yes, a lot,” “Yes, a little,” and “Not at all”). Surveys were sent to the child’s guardians, and the child’s participation in survey responses varied by age. PCC surveys were completed from November 3, 2022, through May 9, 2023.

### Study Design

This study used a nested case-control design among children with confirmed SARS-CoV-2 infection. Case participants were defined as children reporting at least 1 new or ongoing symptom lasting for 1 month or more after infection.^9^ Control participants were defined as children not reporting PCC symptoms. Analysis was restricted to children aged 5 to 17 years whose first RT-PCR–confirmed SARS-CoV-2 infection occurred in-study (ie, no self-reported history of prior infection and serology negative, if serology at enrollment available), who completed their PCC survey 60 days or more from their positive SARS-CoV-2 test and were age-eligible for vaccination per Advisory Committee on Immunization Practices (ACIP) recommendations at the time of their positive test.^26,27^ Children younger than 5 years were not age-eligible for vaccination for part of the study period (July 2021 to May 2023) and were excluded due to very low vaccination rates. Approximately half of the children included in analysis had SARS-CoV-2 sequencing. Omicron variant was inferred for children without sequencing because they were enrolled during a time of Omicron predominance.

### Exposures

The exposure of interest was COVID-19 mRNA vaccination with either BNT162b2 (Pfizer-BioNTech) or mRNA-1273 (Moderna) vaccines. Vaccination dates were self-reported and verified with uploaded images of vaccination cards when available. In Texas and Florida vaccination information could also be verified through medical records or the state immunization information system. Vaccinated status was defined as completion of 2 or more doses of monovalent mRNA COVID-19 vaccine at least 14 days prior to SARS-CoV-2 infection, as recommended at the time of the study. Children with less than 2 vaccine doses were excluded from analysis.

### Outcomes

The outcome of interest was report of any PCC symptoms, defined as any new or ongoing symptoms lasting at least 1 month after the positive SARS-CoV-2 test. PCC symptoms were grouped based on symptoms most frequently reported by study participants and by children in the literature.^28^ PCC symptoms were divided into 2 categories for analysis: respiratory and nonrespiratory (eMethods 1 in Supplement 1).

### Statistical Analysis

Univariate analysis was performed using χ^2^ or Fisher Exact test. Difference in means was assessed with the *t* test or Wilcoxon-Mann-Whitney test when distribution was nonnormal. The adjusted odds ratios (aORs) of PCC among those vaccinated compared with those unvaccinated were estimated using logistic regression. Vaccination effectiveness against development of PCC was estimated as ([1 − aOR] × 100%). Separate multivariable logistic models were created for respiratory, nonrespiratory, at least 1, and at least 2 symptoms as well as for impact of PCC symptoms on function. Models were adjusted for sex (female and male or not reported), age at infection (5-11 years and 12-17 years), number of SARS-CoV-2 symptoms at time of acute infection (0-3 and ≥4), self-rated baseline health (excellent or very good and good, fair, or poor) and time between SARS-CoV-2 infection and PCC survey completion (60-129 days, 130-315 days, and ≥316 days). Age groups were selected based on ACIP age-based vaccine recommendations.^26,27^ Covariates were selected a priori for inclusion based on literature review.^15,16,17,29^ Number of SARS-CoV-2 symptoms during acute infection was used as a proxy for SARS-CoV-2 illness severity as no children were hospitalized for their infections and details of any outpatient care were unknown. Because COVID-19 vaccination is associated with decreased SARS-CoV-2 illness severity (measured as number of symptoms in this analysis) and decreased SARS-CoV-2 illness severity is associated with decreased risk of PCC, adjusting for SARS-CoV-2 illness severity could lead to an underestimate of the effect of COVID-19 vaccination on PCC. However, we reran all models without adjusting for number of SARS-CoV-2 infection symptoms and found similar results (eTable 1 in Supplement 1). For the descriptive analysis, results with a *P* < .05 were considered statistically significant. Odds ratios were considered significant when the calculated confidence interval did not include the value of 1. Analyses were conducted using SAS version 9.4 (SAS Institute).

## Results

During the study period, 1389 children had at least 1 positive SARS-CoV-2 test. After applying analytic sample restrictions described previously, 622 participants were eligible for inclusion (Figure). Of these, 28 (5%) were case participants and 594 (95%) were control participants.

**Figure.**
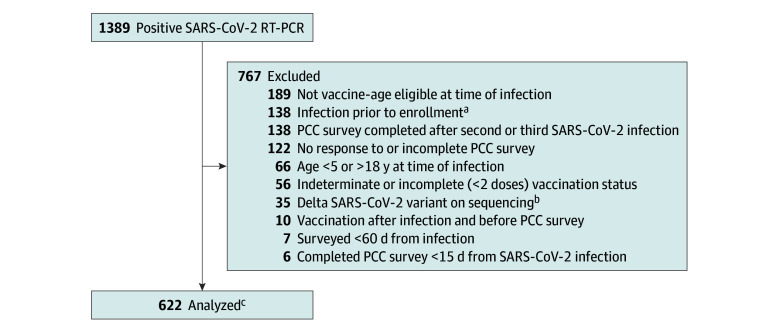
Study Flowchart PCC indicates post–COVID-19 condition; RT-PCR, real time–polymerase chain reaction. ^a^Of 138 persons excluded from analysis for evidence of SARS-CoV-2 infection prior to enrollment, 66 (48%) had self-reported and documented prior positive SARS-CoV-2 test, 51 (37%) had positive SARS-CoV-2 serology testing at enrollment, and 21 (15%) had both. ^b^Thirty-five participants (4 cases, 31 controls) with Delta variant SARS-CoV-2 on sequencing were excluded to focus analysis on PCC after Omicron variant infection where increased risk of more severe COVID-19 due to Delta variant would not be an effect modifier. ^c^Of 622 participants, 269 (43%) had SARS-CoV-2 serology testing. Of these, 158 (59%) were negative and 111 (41%) were positive but obtained after either in-study infection or COVID-19 vaccination so not treated as evidence of prior SARS-CoV-2 infection.

Both case and control participants had a median age of 10 years (with IQRs of 7.0-11.9 and 7.8-12.7 years, respectively). Both groups were approximately 50% female (case participants, 13 [46%]; control participants, 287 [48%]). In terms of race and ethnicity, 4 case participants (14%) were Hispanic, 5 (18%) non-Hispanic Black or African American, and 16 (57%) non-Hispanic White; 156 control participants (26%) were Hispanic, 10 (2%) were non-Hispanic Black or African American, and 357 (60%) were non-Hispanic White, with a higher of proportion of case participants reporting non-Hispanic Black or African American race and ethnicity than control participants (*P* = .001) (Table 1). A higher proportion of cases reported good, fair, or poor baseline health (vs excellent or very good baseline health) compared with controls (7 [25%] vs 54 [9%]; *P* = .01). Although not statistically significant, 4 cases (14%) and 57 controls (10%) were asymptomatic at SARS-CoV-2 diagnosis (*P* = .34). Case and control participants completed their PCC surveys a similar median (IQR) number of weeks after acute SARS-CoV-2 infection: 27 (16-44) weeks for case participants and 26 (17-42) weeks for control participants (*P* = .91). Case and control groups differed on COVID-19 vaccination status: 16 cases (57%) and 458 controls (77%) were vaccinated (*P* = .05). Among vaccinated participants, case participants received their last vaccine dose a median (IQR) of 18 (8-30) weeks prior to SARS-CoV-2 infection and control participants received theirs a median (IQR) of 20 (8-30) weeks prior (*P* = .95).

**Table 1.** Demographic and Clinical Characteristics of Case and Control Participants—PROTECT Cohort, July 2021 to May 2023, 622 Participants^a^

Characteristic	Participants, No. (%)		*P* value
	Cases (n = 28 [5%])^b^	Controls (n = 594 [95%])^b^	
Demographic			
Age at infection onset, median (IQR), y	10.0 (7.0-11.9)	10.3 (7.8-12.7)	.37
Age group at infection onset, y			
5-11	21 (75)	411 (69)	.51
12-17	7 (25)	183 (31)	
Sex^c^			
Female	13 (46)	287 (48)	.88
Male	14 (50)	288 (48)	
Race and ethnicity^d^			
Hispanic	4 (14)	156 (26)	.001
Non-Hispanic Asian	3 (11)	51 (9)	
Non-Hispanic Black or African American	5 (18)	10 (2)	
Non-Hispanic White	16 (57)	357 (60)	
Cohort location			
Phoenix, Arizona	2 (7)	86 (14)	.12
Tucson, Arizona	15 (54)	254 (43)	
Other, Arizona	3 (11)	64 (11)	
Miami, Florida	5 (18)	68 (11)	
Temple, Texas	3 (11)	41 (7)	
Salt Lake City, Utah	0 (0)	81 (14)	
Underlying medical conditions			
Number of chronic conditions			
None	26 (93)	538 (91)	>.99
≥1	2 (7)	56 (9)	
Daily medication use^e^			
None	15 (54)	334 (56)	.77
≥1	13 (46)	258 (43)	
Self-rated health^f^			
Excellent or very good	21 (75)	539 (91)	.01
Good, fair, or poor	7 (25)	54 (9)	
COVID-19 vaccination			
0 Doses^g^	12 (43)	136 (23)	.05
2 Doses^h^	12 (43)	356 (60)	
>2 Doses^i^	4 (14)	102 (17)	
SARS-CoV-2 infection characteristics			
Symptomatic at diagnosis^j^	24 (86)	537 (90)	.34
Asymptomatic at diagnosis	4 (14)	57 (10)	
Self-reported symptoms during infection, No.			
0-3	9 (32)	293 (49)	.08
≥ 4	19 (68)	301 (51)	
Time from positive SARS-CoV-2 test to PCC survey completion, median (IQR), wk	27 (16-44)	26 (17-42)	.91
Time from last COVID-19 vaccine dose to positive SARS-CoV-2 test, median (IQR), wk^k^	18 (8-30)	20 (8-30)	.95

Abbreviation: PCC, post–COVID-19 condition.

^a^ Characteristics are self-reported or parent-reported for participants aged 13 years or younger in Arizona or younger than 17 years in Florida, Texas, and Utah. Case participants are children who reported symptoms associated with PCC; control participants are those who did not.

^b^ PCC defined as at least 1 new or ongoing symptom lasting at least 1 month after infection; see eMethods 1 in Supplement 1 for full list of PCC symptoms.

^c^ Not reported for 1 case participant and 19 control participants.

^d^ No participants self-identified as non-Hispanic American Indian or Alaskan Native; non-Hispanic Asian category includes persons who responded as native Hawaiian or Other Pacific Islander or multiracial; 20 persons in the control group did not disclose race or ethnicity.

^e^ Two persons in the control group did not disclose medication use.

^f^ One person in the control group did not disclose self-rated health.

^g^ No record of receipt of any mRNA COVID-19 vaccine at least 7 days prior to SARS-CoV-2 infection.

^h^ Receipt of 2 mRNA COVID-19 doses at least 14 days prior to SARS-CoV-2 infection.

^i^ Receipt of more than 2 mRNA COVID-19 doses at least 7 days prior to SARS-CoV-2 infection.

^j^ One or more COVID-19–like illness symptoms.

^k^ Time since last COVID-19 vaccine among vaccinated participants only.

Nearly all vaccinated children (470 of 474 [99%]) received the BNT162b2 vaccine (Table 2). Vaccinated children were older than unvaccinated children (median [IQR] age, 10.5 [7.9-13.0] years vs 9.7 [7.4-11.7] years; *P* = .005), and a higher proportion were female, although this difference was not statistically significant (236 [50%] vs 64 [43%]; *P* = .07). A higher proportion of vaccinated children were non-Hispanic Asian (46 [10%]) and non-Hispanic White (290 [61%]) compared with unvaccinated children (8 [5%] and 83 [56%], respectively]) (*P* = .004). Vaccinated children were more likely than unvaccinated children to reside in Tucson, Arizona (231 [49%] vs 38 [26%]), Miami, Florida (38 [8%] vs 35 [24%]), and Temple, Texas (26 [5%] vs 18 [12%]) (*P* < .001). A higher proportion of vaccinated children than unvaccinated children reported having 4 or more symptoms during acute SARS-CoV-2 infection (vs 0-3) (257 [54%] vs 63 [43%]; *P* = .01), had no daily medication use (280 [59%] vs 69 [47%]; *P* = .007), and reported excellent or very good baseline health (vs good, fair, or poor) (436 [92%] vs 124 [84%]; *P* = .007).

**Table 2.** Demographic and Clinical Characteristics of Vaccinated and Unvaccinated Children—PROTECT Cohort, July 2021 to May 2023, 622 Participants^a^

Characteristic	Participants, No. (%)		*P* value
	Vaccinated (n = 474)^b^	Unvaccinated (n = 148)	
Demographic			
Age at infection onset, median (IQR), y	10.5 (7.9-13.0)	9.7 (7.4-11.7)	.005
Age group at infection onset, y			
5-11	314 (66)	118 (80)	.002
12-17	160 (34)	30 (20)	
Sex^c^			
Female	236 (50)	64 (43)	.07
Male	219 (46)	83 (56)	
Race and ethnicity^d^			
Hispanic	112 (24)	48 (32)	.004
Non-Hispanic Asian	46 (10)	8 (5)	
Non-Hispanic Black or African American	7 (1)	8 (5)	
Non-Hispanic White	290 (61)	83 (56)	
Cohort location			
Phoenix, Arizona	74 (16)	14 (9)	<.001
Tucson, Arizona	231 (49)	38 (26)	
Other, Arizona	44 (9)	23 (16)	
Miami, Florida	38 (8)	35 (24)	
Temple, Texas	26 (5)	18 (12)	
Salt Lake City, Utah	61 (13)	20 (14)	
Underlying medical conditions			
No. of chronic conditions			
None	424 (89)	140 (95)	.06
≥1	50 (11)	8 (5)	
Daily medication use^e^			
None	280 (59)	69 (47)	.007
≥1	192 (41)	79 (53)	
Self-rated health^f^			
Excellent or very good	436 (92)	124 (84)	.007
Good, fair, or poor	38 (8)	23 (16)	
Vaccine product			
BNT162b2 (Pfizer-BioNTech)	470 (99)	NA	NA
mRNA-1273 (Moderna)	2 (<1)	NA	
Mixed dosing	2 (<1)	NA	
SARS-CoV-2 infection characteristics			
Symptomatic at diagnosis^g^	440 (93)	121 (82)	<.001
Asymptomatic at diagnosis	34 (7)	27 (18)	
Self-reported symptoms during infection, No.			
0-3	217 (46)	85 (57)	.01
≥4	257 (54)	63 (43)	

Abbreviation: NA, not applicable.

^a^ Characteristics are self-reported or parent-reported for participants aged 13 years or younger in Arizona or younger than 17 years of age in Florida, Texas, and Utah.

^b^ Vaccinated defined as completion of at least 2 doses of monovalent mRNA COVID-19 vaccine as recommended at the time of the study at least 14 days prior to SARS-CoV-2 infection.

^c^ Not reported for 19 participants in the vaccinated group and 1 participant in the unvaccinated group.

^d^ No participants self-identified as non-Hispanic American Indian or Alaska Native; the non-Hispanic Asian category includes individuals who responded as Native Hawaiian or Other Pacific Islander or multiracial; 19 persons in the vaccinated group and 1 person in the unvaccinated group did not disclose race or ethnicity.

^e^ Two persons in the vaccinated group did not disclose medication use.

^f^ One person in the unvaccinated group did not disclose self-rated health.

^g^ One or more COVID-19–like illness symptoms.

The aOR of 1 or more PCC symptoms in vaccinated vs unvaccinated children was 0.43 (95% CI, 0.19-0.98), and the aOR of 2 or more PCC symptoms was 0.27 (95% CI, 0.10-0.69) (Table 3). The aOR of respiratory PCC symptoms was 0.28 (95% CI, 0.10-0.75), and the aOR of nonrespiratory PCC symptoms was 0.49 (95% CI, 0.20-1.21). Of the 28 case participants reporting at least 1 PCC symptom, 16 (57%) reported PCC symptoms impacting function. The aOR of reporting PCC symptom impact on function was 0.25 (95% CI, 0.08-0.74).

**Table 3.** Adjusted Odds of PCC Symptoms in Vaccinated Children Compared With Unvaccinated Children—PROTECT Cohort, July 2021 to May 2023, 615 Participants^a^

Outcome	Total (n = 615), No. (column %)	No. (row %)		OR (95% CI)	Adjusted OR (95% CI)^c^
		Unvaccinated (n = 147)	Vaccinated (n = 468)^b^		
No PCC symptoms (control participants)	587 (95)	135 (23)	452 (77)	1 [Reference]	1 [Reference]
≥1 PCC symptom^d^	28 (5)	12 (43)	16 (57)	0.40 (0.18-0.86)	0.43 (0.19-0.98)
≥2 PCC symptoms^d^	21 (3)	11 (52)	10 (48)	0.27 (0.11-0.65)	0.27 (0.10-0.69)
Respiratory PCC symptoms^d^	19 (3)	10 (53)	9 (47)	0.27 (0.11-0.68)	0.28 (0.10-0.75)
Nonrespiratory PCC symptoms	24 (4)	10 (42)	14 (58)	0.42 (0.18-0.96)	0.49 (0.20-1.21)
PCC impact on function, No/total No. (%)^e^	16/65 (57)	9/16 (56)	7/16 (44)	0.23 (0.08-0.64)	0.25 (0.08-0.74)

Abbreviations: OR, odds ratio; PCC, post–COVID-19 condition.

^a^ Seven control participants with missing covariate values excluded (1 unvaccinated, 6 vaccinated).

^b^ Vaccinated defined as completion of at least 2 doses of monovalent mRNA COVID-19 vaccine as recommended at the time of the study at least 14 days prior to SARS-CoV-2 infection.

^c^ Adjusted OR estimated using logistic regression with unvaccinated as referent group adjusted for sex, age at infection, number of symptoms experienced during infection, self-rated health at baseline, and time between infection and survey completion.

^d^ Symptoms include: respiratory (shortness of breath, runny nose or nasal congestion, and cough) and nonrespiratory (fever, unexplained weight loss, unexplained weight gain, symptoms that get worse after physical activity, change in general physical levels, “brain fog,” fatigue, change in sleeping, leg swelling, hair loss, change in color of finger or toes, rash, bruising or bleeding easily, palpitations, chest pain or tightness, dizziness, numbness, headache, difficulty speaking or communicating, difficulty swallowing or chewing, problems with balance, memory loss, difficulty concentrating, nerve problems [tremors, shaking, abnormal movement, new seizures], problem with hearing loss or ears ringing, joint pain, joint swelling, muscle pain, loss of appetite, increased appetite, change in taste, change in smell, nausea, vomiting, abdominal or stomach pain, constipation, diarrhea, bloating, and bladder problems).

^e^ Interviewees asked “Do any PCC reduce your child’s ability to carry-out day-to-day activities, including attending school and participating in activities and sports?” with answer options “Yes, a lot,” “Yes, a little,” and “Not at all.” Adjusted OR was calculated for “Yes, a lot” and “Yes, a little” combined.

## Discussion

This study found that COVID-19 vaccination prior to SARS-CoV-2 Omicron infection was associated with a 57% reduced likelihood of 1 or more PCC symptoms, a 73% reduced likelihood of 2 or more PCC symptoms, and a 72% reduced likelihood of respiratory PCC symptoms among children aged 5 to 17 years. In addition, COVID-19 vaccination prior to SARS-CoV-2 Omicron infection was associated with a 75% reduced likelihood of PCC symptoms impacting day-to-day function. Because both case and control participants had SARS-CoV-2 infection, the overall protection against PCC from vaccination is likely even higher, as these estimates do not account for prevention of SARS-CoV-2 infection by vaccination.^30,31,32^ These data showing reduced odds of PCC in children with vaccination are consistent with findings in adults and limited findings in children that show that COVID-19 vaccination is associated with lower risk of PCC.^15,18,19,20,28^

The CDC’s nationwide pediatric COVID-19 seroprevalence surveillance shows that an estimated 96% of children are seropositive for SARS-CoV-2 and that over 65 million children in the US have had SARS-CoV-2 infection as of January 2023.^3^ This suggests that nearly all US children will experience at least 1 SARS-CoV-2 infection. Although children generally experience mild or asymptomatic SARS-CoV-2 infection, they are still at risk for developing PCC which can carry significant morbidity.

The data from this study are important as they indicate that even in a population that typically experiences only mild COVID-19, vaccination is associated with lower odds of PCC. Pediatric COVID-19 vaccine uptake in the US has been relatively low, particularly in younger children.^33^ According to weekly national immunization survey data, only 12% of children 5 to 17 years of age were up to date with the updated 2024-2025 COVID-19 vaccine as of December 2024.^34^ Surveys have shown that one reason behind parental COVID-19 vaccine hesitancy is the idea that COVID-19 in children is usually a mild illness and therefore vaccination is not necessary.^35,36^ However, even mild or asymptomatic SARS-CoV-2 infection can result in postinfectious sequelae.^37,38^ Several children with PCC in this cohort had asymptomatic SARS-CoV-2 infections at diagnosis (although they may have gone on to develop symptoms), and none had an infection that resulted in hospitalization, suggesting that although severe SARS-CoV-2 illness is a risk factor for PCC,^12,15,17^ PCC may develop without severe or even symptomatic COVID-19 illness. We found that a higher proportion of non-Hispanic Black or African American children reported PCC. This finding should be interpreted with caution due to small sample size but is consistent with data from earlier in the COVID-19 pandemic showing that Hispanic and non-Hispanic Black individuals were at higher risk of COVID-19 hospitalization and severe COVID-19, both risk factors for PCC.^39,40^ We also found that a higher proportion of case participants reported good, fair, or poor baseline health (as opposed to excellent or very good health), suggesting that participants with poorer baseline health may be more likely to report PCC. Poorer baseline health could be attributed to underlying conditions, although only 2 case participants (7%) and 56 control participants (9%) reported having 1 or more underlying conditions. Poorer baseline health could also be related to other factors, such as access to health care or food insecurity.^41^ Several studies have found that certain underlying conditions, such as allergies, atopic dermatitis, and asthma, are risk factors for PCC in children and adults.^16,42,43^ Odds estimates in this analysis could not be adjusted for presence of specific underlying conditions as few children in this cohort reported presence of any underlying conditions. The association between particular underlying conditions in children and risk of PCC is an area needing further study.

Vaccine uptake in our cohort was higher than the national average but followed national trends: a higher proportion of older children were vaccinated and more non-Hispanic Asian and White children were vaccinated compared with Hispanic children of any race and non-Hispanic Black children.^44,45^ Increased vaccine uptake in our cohort is likely due to multiple factors, including the fact that many participants were the children of health care workers, first responders, and other essential workers who were themselves enrolled in other research cohorts.^24^ Although patterns in vaccine uptake in this cohort should be interpreted with caution given small sample size, they do highlight the continued need for vaccine equity among children belonging to racial and ethnic minority groups.^46^

### Limitations

This study is subject to several limitations. Participants may have been misclassified according to infection status prior to enrollment as not all participants agreed to serology testing at enrollment and some had serology testing obtained after COVID-19 vaccination (that did not distinguish anti-spike from anti-nucleocapsid antibodies) or after documented in-study infection. Participants may have misclassified symptoms of acute SARS-CoV-2 infection, symptoms from underlying conditions, or symptoms from other illnesses as PCC symptoms. In this cohort, vaccinated individuals reported a larger number of COVID-19 symptoms compared with unvaccinated individuals. Vaccinated individuals in this cohort may be more likely to report symptoms (both SARS-CoV-2 infection and PCC symptoms) due to participation bias. Vaccinated individuals being more likely to report PCC symptoms could lead to an underestimate of protection provided by vaccination. As participants were only asked about SARS-CoV-2 symptoms at time of positive test, some who reported no symptoms and were classified as asymptomatic may have gone on to develop symptoms. Generalizability of results for this analysis is limited by small sample size, a largely non-Hispanic White population, and the cohort being predominantly children of health care and frontline workers. Children of health care and frontline workers may have better health care access and health literacy compared with the general population, leading to an increased reporting of PCC symptoms. As the cohort was majority non-Hispanic White, we were not powered to adjust for race and ethnicity, which may bias results. Use of the PCC definition of 4 weeks or longer may have led to the identification of more children with PCC than would have been identified with the NASEM definition. Slightly more than 10% of participants were lost to follow-up. While those lost to follow-up did not have a distinct characteristic profile differing from those who completed the study (eTable 2 in Supplement 1), it is unknown whether participants lost to follow-up were more or less likely to experience PCC. Findings are limited to the BNT162b2 COVID-19 vaccine, given that 99% of vaccinated participants received BNT162b2.

## Conclusions

This study found that COVID-19 vaccination prior to SARS-CoV-2 Omicron infection was associated with 57% to 73% reduced likelihood of PCC symptoms in children and 75% reduced likelihood of decreased function from PCC symptoms. Our findings suggest that children should stay up to date with current COVID-19 vaccination recommendations as vaccination not only protects against severe COVID-19 illness but also protects against PCC.^47^

## Notes

Supplement 1. **eMethods 1.** Definition of Symptoms Included in Respiratory and Nonrespiratory Categories **eTable 1.** Estimate of Adjusted Odds of PCC Symptoms in Vaccinated Children Compared With Unvaccinated Children With Number of SARS-CoV-2 Symptoms and Time From SARS-CoV-2 Infection to PCC Survey Covariates Excluded **eTable 2.** Characteristics of Participants Included in Analysis and Those Lost to Follow-Up **eMethods 2.** Pediatric Research Observing Trends and Exposures in COVID-19 Timelines (PROTECT) Long-COVID (PCC) Questionnaire

Supplement 2.
Data Sharing Statement
