# Supplementary material for: COVID-19 Vaccination and Odds of Post–COVID-19 Condition Symptoms in Children Aged 5 to 17 Years
Source: JAMA Netw Open. 2025 Feb 24;8(2):e2459672. doi: 10.1001/jamanetworkopen.2024.59672 (PMC11851240; doi:10.1001/jamanetworkopen.2024.59672)
Supplement: Supplement 1. — eMethods 1. Definition of Symptoms Included in Respiratory and Nonrespiratory Categories eTable 1. Estimate of Adjusted Odds of PCC Symptoms in Vaccinated Children Compared With Unvaccinated Children With Number of SARS-CoV-2 Symptoms and Time From SARS-CoV-2 Infection to PCC Survey Covariates Excluded eTable 2. Characteristics of Participants Included in Analysis and Those Lost to Follow-Up eMethods 2. Pediatric Research Observing Trends and Exposures in COVID-19 Timelines (PROTECT) Long-COVID (PCC) Questionnaire [file jamanetwopen-e2459672-s001.pdf]

Supplemental Online Content

Yousaf AR, Mak J, Gwynn L, et al. COVID-19 vaccination and odds of post–COVID-19 condition symptoms in children aged 5 to 17 years. *JAMA Netw Open.* 2025;8(2):e2459672. doi:10.1001/jamanetworkopen.2024.59672

eMethods 1. Definition of Symptoms Included in Respiratory and Nonrespiratory Categories

eTable 1. Estimate of Adjusted Odds of PCC Symptoms in Vaccinated Children Compared With Unvaccinated Children With Number of SARS-CoV-2 Symptoms and Time From SARS-CoV-2 Infection to PCC Survey Covariates Excluded

eTable 2. Characteristics of Participants Included in Analysis and Those Lost to Follow-Up

eMethods 2. Pediatric Research Observing Trends and Exposures in COVID-19 Timelines (PROTECT) Long-COVID (PCC) Questionnaire

Data Sharing Statement

This supplemental material has been provided by the authors to give readers additional information about their work.

© 2025 Yousaf AR et al. *JAMA Network Open*.

eMethods 1. Definition of Symptoms Included in Respiratory and Nonrespiratory Categories

Respiratory: shortness of breath, runny nose or nasal congestion, cough

Non-Respiratory: fever, unexplained weight loss or weight gain, exertional malaise, change in energy, fatigue, dizziness, "brain fog", change in sleep pattern, numbness, headache, difficulty speaking or communicating, difficulty swallowing or chewing, problems with balance, memory loss, difficulty concentrating, nerve problems [tremors, shaking, abnormal movement, new seizures], problems with hearing loss or ears ringing, change in taste or smell, palpitations, chest pain or tightness, loss of appetite, increased appetite, nausea, vomiting, abdominal pain, constipation, diarrhea, bloating, joint pain or swelling, muscle pain, leg swelling, hair loss, change in color of fingers or toes, rash, bruising or bleeding easily, and bladder problems

© 2025 Yousaf AR et al. *JAMA Network Open*.

eTable 1. Estimate of Adjusted Odds of PCC Symptoms in Vaccinated Children Compared With Unvaccinated Children With Number of SARS-CoV-2 Symptoms and Time From SARS-CoV-2 Infection to PCC Survey Covariates Excluded, N=615[a]

	Total N=615 n (column %)	Unvaccinated, N=147 n (row %)	Vaccinated[b], 468 n (row %)	OR (95% CI)	Adjusted OR (95% CI)[c]
No PCC symptoms (controls)	587 (95)	135 (23)	452 (77)	ref	ref
1 or more PCC symptoms[d]	28 (5)	12 (43)	16 (57)	0.40 (0.18 - 0.86)	0.46 (0.21 - 1.03)
2 or more PCC symptoms[d]	21 (3)	11 (52)	10 (48)	0.27 (0.11 - 0.65)	0.31 (0.13 - 0.77)
Respiratory PCC symptoms[d]	19 (3)	10 (53)	9 (47)	0.27 (0.11 - 0.68)	0.31 (0.12 - 0.80)
Non-Respiratory PCC symptoms[d]	24 (4)	10 (42)	14 (58)	0.42 (0.18 - 0.96)	0.51 (0.21 - 1.21)
PCC Impact on function[e]	16 (3)	9 (56)	7 (44)	0.23 (0.08 - 0.64)	0.27 (0.09 - 0.78)

Abbreviations: CI = confidence interval; OR=Odds Ratio

[a]Seven controls with missing covariate values excluded (1 unvaccinated, 6 vaccinated)

[b]Vaccinated defined as completion of at least 2 doses of monovalent mRNA COVID-19 vaccine as recommended at the time of the study ≥14 days prior to SARS-CoV-2 infection.

[c]Adjusted odds ratio estimated using logistic regression with unvaccinated as referent group adjusted for sex, age at infection, and self-rated health.

[d]Symptoms include: respiratory (shortness of breath, runny nose or nasal congestion, and cough), and non-respiratory (fever, unexplained weight loss, unexplained weight gain, symptoms that get worse after physical activity, change in general physical levels, “brain fog”, fatigue, change in sleeping, leg swelling, hair loss, change in color of finger or toes, rash, bruising or bleeding easily, palpitations, chest pain or tightness, dizziness, numbness, headache, difficulty speaking or communicating, difficulty swallowing or chewing, problems with balance, memory loss, difficulty concentrating, nerve problems [tremors, shaking, abnormal movement, new seizures], problem with hearing loss or ears ringing, joint pain, joint swelling, muscle pain, loss of appetite, increased appetite, change in taste, change in smell, nausea, vomiting, abdominal or stomach pain, constipation, diarrhea, bloating, bladder problems).

[e]Interviewees asked “Do any PCC reduce your child’s ability to carry-out day-to-day activities, including attending school and participating in activities and sports?” with answer options “Yes, a lot”, “Yes, a little”, and “Not at all”. aOR calculated for “Yes, a lot” and “Yes, a little” combined.

© 2025 Yousaf AR et al. *JAMA Network Open*.

eTable 2. Characteristics of Participants Included in Analysis and Those Lost to Follow-Up

	Included in Analysis<br>N=622<br>n (%)	Lost to Follow-up[a]<br>N=178<br>n (%)
Demographic		
Age at infection onset, median (IQR), y	10.0 (7.0 - 11.9)	10.6 (7.7 – 12.8)
Age group at infection onset, y		
5-11	432 (69)	121 (68)
12-17	190 (31)	57 (32)
Sex[b]		
Female	300 (48)	86 (48)
Male	302 (49)	83 (47)
Race and ethnicity[c]		
Hispanic	160 (26)	57 (32)
Non-Hispanic Asian	54 (9)	19 (11)
Non-Hispanic American Indian/Alaskan Native[d]	0 (0)	1 (1)
Non-Hispanic Black or African American	15 (2))	0 (0)
Non-Hispanic White	373 (60)	90 (51)
Cohort location		
Phoenix, Arizona	88 (14)	36 (20)
Tucson, Arizona	269 (43)	99 (56)
Other, Arizona	67 (11)	16 (9)
Miami, Florida	73 (12)	11 (6)
Temple, Texas	44 (7)	8 (4)
Salt Lake City, Utah	81 (13)	8 (4)
Underlying medical conditions		
Number of chronic conditions		
None	564 (91)	153 (86)
≥1	58 (9)	25 (14)
Daily medication use[e]		
None	349 (56)	115 (65)
≥1	271 (44)	53 (30)
Self-rated health[f]		
Excellent/very good	560 (90)	154 (87)
Good/fair/poor	61 (10)	22 (12)
SARS-CoV-2 infection characteristics		
Symptomatic at diagnosis[g]	561 (90)	169 (95)
Asymptomatic at diagnosis	61 (10)	9 (5)
Number of self-reported symptoms during infection		
0-3	302 (49)	97 (54)
≥4	320 (51)	81 (46)

[a]Individuals who did not respond to the Post-COVID Condition (PCC) survey, responded to the PCC survey but did not report any specific symptom(s), had an indeterminate vaccination status (<7 days between acute infection and when vaccine was received), or less than 2 vaccine doses at the time of acute infection.
[b]Not reported for 20 participants in the analytic sample and 9 in the no response group.

© 2025 Yousaf AR et al. *JAMA Network Open*.

[c]Twenty participants in the “analytic sample” group, and 11 in the “no response” group did not disclose their race and ethnicity.
[d]Non-Hispanic Asian category includes persons who responded as native Hawaiian/other Pacific Islander or Multiracial.
[e]Daily medication defined as physician prescribed medication. Two participants in the “analytic sample” group and 10 in the “no response” group did not disclose their current daily medication use.
[f]One participant in the “analytic sample” group and 2 in the “no response” group did not disclose their self-rated health.
[g]Symptomatic defined as self-report of at least one COVID-like illness symptom.

© 2025 Yousaf AR et al. *JAMA Network Open*.

eMethods 2. Pediatric Research Observing Trends and Exposures in COVID-19 Timelines (PROTECT) Long-COVID (PCC) Questionnaire

Administrative Information

1. Study Site: University of Arizona ☐ BSWH ☐ University of Miami ☐ University of UT
2. Participant ID: ______________________
3. Participant Last Name _________________
4. Participant First Name_________________
5. Participant DOB______________________

This form will be administered to all participants 60 days after a recorded positive COVID-19 test. For influenza cases and illness cases, this form may be administered to all or a defined subset of participants 60 days after a recorded positive influenza test or illness onset. The survey will be re-administered every 180 days after the previous survey (to ask about the month prior).

[INTRO- 60 days after illness]

Thank you for taking the time to complete this survey. We are adding this new survey to learn more about the impact of long-term illness on people's health. This survey will take about 5 minutes to complete, and we may be resending this survey in 6 months.

[INTRO- 180 days after previous survey]

Thank you for taking the time to complete this survey to help us learn more about the impact of long-term illness on people's health. This survey will take about 5 minutes to complete.

[FOR COVID-19 CASES]

During the PROTECT Study, we recorded a positive COVID-19 test for [Child's Name] on [DATE]. These survey questions ask about the time period since [DATE].

[FOR INFLUENZA CASES]

During your participation in the PROTECT Study, we recorded an influenza infection for [Child's Name] on x/x/x. These survey questions ask about the time period since [DATE].

[FOR OTHER ILLNESS CASES]

During your participation in the PROTECT Study, we recorded an illness for [Child's Name]. These survey questions ask about the time period since the start of their symptoms which occurred on [DATE].

A. SYMPTOMS

A1. Since your child was ill on [DATE], have they had any symptoms for any illness that lasted longer than 1 month?

a. Yes → **CONTINUE TO A2**
b. No → **END OF SURVEY**

A2. Please tell us if they had any of the symptoms below for **more than a month after their illness.**

© 2025 Yousaf AR et al. *JAMA Network Open*.

These symptoms are grouped by areas of the body. Please check all that apply. → IF NONE SELECTED SKIP TO A3

A2a. General

☐ Fever

☐ Unexplained weight loss

☐ Unexplained weight gain, for example weight gain beyond what is expected for their age or height

☐ Symptoms that get worse after physical activity (e.g., walking stairs, playing a sport)

☐ Change in general physical activity levels

☐ Difficulty or fogginess in thinking or concentrating (sometime referred to as "brain fog")

☐ Fatigue/unusual tiredness/weakness

☐ Change in sleeping (e.g., not able to sleep through the night, poor sleep)

☐ Leg swelling

A2b. Skin / Hair

☐ Hair loss

☐ Change in color of toes / fingers

☐ Rash

☐ Bruising / bleeding easily

A2c. Heart / Lungs

☐ Palpitations (heart beating fast or irregular)

☐ Chest pain / chest tightness

☐ Shortness of breath

☐ Runny nose / nasal congestion

☐ Cough

A2d.Brain / Nerves

☐ Dizziness / lightheadedness / fainting

☐ Numbness / tingling

☐ Headache

☐ Difficulty speaking / communicating

☐ Difficulty swallowing / chewing

☐ Problems with balance / movement

☐ Memory loss

☐ Difficulty concentrating

☐ Nerve problems (tremors, shaking, abnormal movement, new seizures)

☐ Problems with hearing loss or ears ringing

A2e. Muscle / Joint

☐ Joint pain

☐ Joint swelling

☐ Muscle pain

© 2025 Yousaf AR et al. *JAMA Network Open*.

A2f. Stomach / Intestines

☐ Loss of appetite
☐ Increased appetite
☐ Change in taste
☐ Change in smell
☐ Nausea
☐ Vomiting
☐ Abdominal / stomach pain
☐ Constipation
☐ Diarrhea
☐ Bloating
☐ Bladder problems (can't hold in urine or trouble passing urine)

A2g. Since your child's illness, have they received a diagnosis for any of the following conditions:

☐ Multisystem inflammatory syndrome (MIS-C)
☐ Kawasaki Disease
☐ Myocarditis
☐ Other → CONTINUE TO A2h

A2h. Please note any other condition or diagnoses your child received.
[FREE TEXT BOX]

A3. Did your child experience any other symptoms which were not listed above?

☐ Yes → **CONTINUE TO A3a**
☐ No → **CONTINUE TO A4**

A3a. Please note any other symptoms your child may have experienced which are not listed above:

[FREE TEXT BOX]

A4. Below are the symptoms reported from the time of illness. Starting from the time of infection, please indicate how long the symptoms lasted or if they are still ongoing.

(SYMPTOMS AUTOPOPULATE FROM QUESTION A2)

	Symptoms ongoing	lasted 1 to 2 months	Lasted 3-4 months	Lasted 5-6 months	Lasted more than 6 months
Symptom 1	○	○	○	○	○
Symptom 2	○	○	○	○	○
Symptom 3	○	○	○	○	○
Symptom 4	○	○	○	○	○
Etc.	○	○	○	○	○

© 2025 Yousaf AR et al. *JAMA Network Open*.

A5. For any of the symptoms listed below, were your child's daily activities impacted? (e.g., an activity took longer than usual or was harder to complete) (CHECKBOX)
(SYMPTOMS AUTOPOPULATE FROM QUESTION A2)

	Yes (CHECKBOX)	No (CHECKBOX)	Not Sure (CHECKBOX)
Symptom 1	◯	◯	◯
Symptom 2	◯	◯	◯
Symptom 3	◯	◯	◯
Symptom 4	◯	◯	◯
Etc.	◯	◯	◯

B. FUNCTION

B1. Do any of any of the conditions, symptoms, or illnesses reported reduce your child's ability to carry-out day-to-day activities, including attending school and participating in activities and sports? *(Select one)*

☐ Yes, a lot → **Continue to B2**
☐ Yes, a little → **Continue to B2**
☐ Not at all → **End Survey**

B2. Think about the time since infection. For about how many days did poor physical or mental health keep your child from doing their usual activities, such as attending school, self-care, work, or recreation?

☐ I can indicate the number of days → **CONTINUE B2a**
☐ Don't Know → **CONTINUE TO End of survey?**
☐ Prefer not to answer → **CONTINUE TO End of survey?**

B2a. Please indicate how many days poor **physical** health kept your child from doing their usual activities, such as school, self-care, work, or recreation?

[FREE TEXT BOX]

B2b. Please indicate how many days poor **mental** health kept your child from doing your usual activities, such as school, self-care, work, or recreation?

[FREE TEXT BOX]

END OF SURVEY

Closing message: Thank you for taking the survey. We may ask you these questions again in 6 months. Have a nice day!

© 2025 Yousaf AR et al. *JAMA Network Open*.
