# Supplementary material for: COVID-19 Vaccination and Odds of Post–COVID-19 Condition Symptoms in Children Aged 5 to 17 Years
Source: JAMA Netw Open. 2025 Feb 24;8(2):e2459672. doi: 10.1001/jamanetworkopen.2024.59672 (PMC11851240; doi:10.1001/jamanetworkopen.2024.59672)
Supplement: Supplement 2. — Data Sharing Statement [file jamanetwopen-e2459672-s002.pdf]

Data Sharing Statement

Yousaf. COVID-19 Vaccination and Odds of Post–COVID-19 Condition Symptoms in Children Aged 5 to 17 Years. *JAMA Netw Open*. Published February 24, 2025. doi:10.1001/jamanetworkopen.2024.59672

Data
Data available: Yes
Data types: Data (not involving human participants)
How to access data: Aggregated, de-identified data will be made available upon request. pgy6@cdc.gov
When available: With publication

Supporting Documents
Document types: None

Additional Information
Who can access the data: Anyone requesting data
Types of analyses: Any specified purpose
Mechanisms of data availability: Without investigator support
